# Glycogen synthase kinase 3β suppresses polyglutamine aggregation by inhibiting Vaccinia-related kinase 2 activity

**DOI:** 10.1038/srep29097

**Published:** 2016-07-05

**Authors:** Eunju Lee, Hye Guk Ryu, Sangjune Kim, Dohyun Lee, Young-Hun Jeong, Kyong-Tai Kim

**Affiliations:** 1 Division of Integrative Biosciences and Biotechnology, Pohang University of Science and Technology, Pohang 790-784, Republic of Korea; 2 Department of Life Sciences, Pohang University of Science and Technology, Pohang 790-784, Republic of Korea; 3 Neuroregeneration and Stem Cell Programs, Institute for Cell Engineering, The johns Hopkins University School of Medicine, Baltimore, Maryland 21205, United States of America; 4 Department of Neurology, The Johns Hopkins University School of Medicine, Baltimore, Maryland 21205, United States of America

## Abstract

Huntington’s disease (HD) is a neurodegenerative disorder caused by an abnormal expansion of polyglutamine repeats in the N-terminal of huntingtin. The amount of aggregate-prone protein is controlled by various mechanisms, including molecular chaperones. Vaccinia-related kinase 2 (VRK2) is known to negatively regulate chaperonin TRiC, and VRK2-facilitated degradation of TRiC increases polyQ protein aggregation, which is involved in HD. We found that VRK2 activity was negatively controlled by glycogen synthase kinase 3β (GSK3β). GSK3β directly bound to VRK2 and inhibited the catalytic activity of VRK2 in a kinase activity-independent manner. Furthermore, GSK3β increased the stability of TRiC and decreased the formation of HttQ103-GFP aggregates by inhibiting VRK2. These results indicate that GSK3β signaling may be a regulatory mechanism of HD progression and suggest targets for further therapeutic trials for HD.

Huntington’s disease (HD) is a neurodegenerative disease caused by abnormal CAG triplet repeats (>35 residues) in the huntingtin (Htt) gene exon 1 at the N-terminal^1^. This expanded polyglutamine repeat causes protein aggregation and progressive cell death^2^. The number of glutamine residues correlates with the severity of HD symptoms and the age of disease onset^3^,^4^. Because the pathological hallmark of HD is the formation of polyQ-containing Htt aggregates, it is important to prevent this process. The level of aggregated protein is controlled by diverse mechanisms such as molecular chaperones^4^,^5^. In particular, the eukaryotic chaperonin TRiC (TCP-1 Ring Complex, also known as CCT for chaperonin containing TCP-1) attenuates Htt-polyQ protein aggregation and reduces cytotoxicity^6^.

The Vaccinia-related kinase (VRK) family is a serine/threonine kinases that is related to the casein kinase I family^7^. VRK2 has two isoforms: VRK2A and VRK2B. VRK2A has a transmembrane domain and localizes mainly in the endoplasmic reticulum, whereas VRK2B lacks a transmembrane domain and localizes mainly in the cytosol and nucleus^8^. So far, identified substrates of VRK2 include NFAT-1 and USP25^9^,^10^. However, because VRK2 substrates are rarely identified, VRK2 function remains largely unknown. Meanwhile, several reports indicate that VRK2 is associated with neurological disorders such as epilepsy^11^, schizophrenia^12^,^13^, and HD^9^,^14^. We previously found that VRK2 downregulates CCT4, which results in increased polyQ aggregation^9^,^14^.

Glycogen synthase kinase 3 (GSK3) is a constitutively active serine/threonine kinase with two isoforms: GSK3α and GSK3β^15^. Its kinase activity is regulated by inhibitory phosphorylation sites at serine-21 and serine-9 in GSK3α and GSK3β, respectively^16^. GSK3β localizes predominantly in the cytoplasm^17^ but is sometimes found in the nucleus^18^, and its subcellular localization changes in response to binding partners or stimuli. GSK3β has several substrates and is involved in many cellular processes, including cell development^19^, proliferation, cell migration^20^, glucose regulation, and apoptosis^18^.

Consistent with its involvement in a variety of signaling pathways, GSK3β is associated with many diseases such as Alzheimer’s disease^21^, cancer^22^,^23^, bipolar disorder^24^, diabetes^22^, and HD. Decreased GSK3β levels and activity are observed in the brains of R6/1 mice, an animal model of HD^25^, and reduced GSK3β levels are also found in the brains of human patients with HD^26^. However, the precise role of GSK3β in HD has not been elucidated.

In this study, we show that GSK3β directly interacted with VRK2 and inhibited VRK2 catalytic activity *in vitro*. Subsequently, GSK3β reduced VRK2-mediated degradation of TRiC, thereby suppressing polyQ-expanded Htt aggregation.

## Results

### GSK3β directly interacts with VRK2

**Figure 1:**
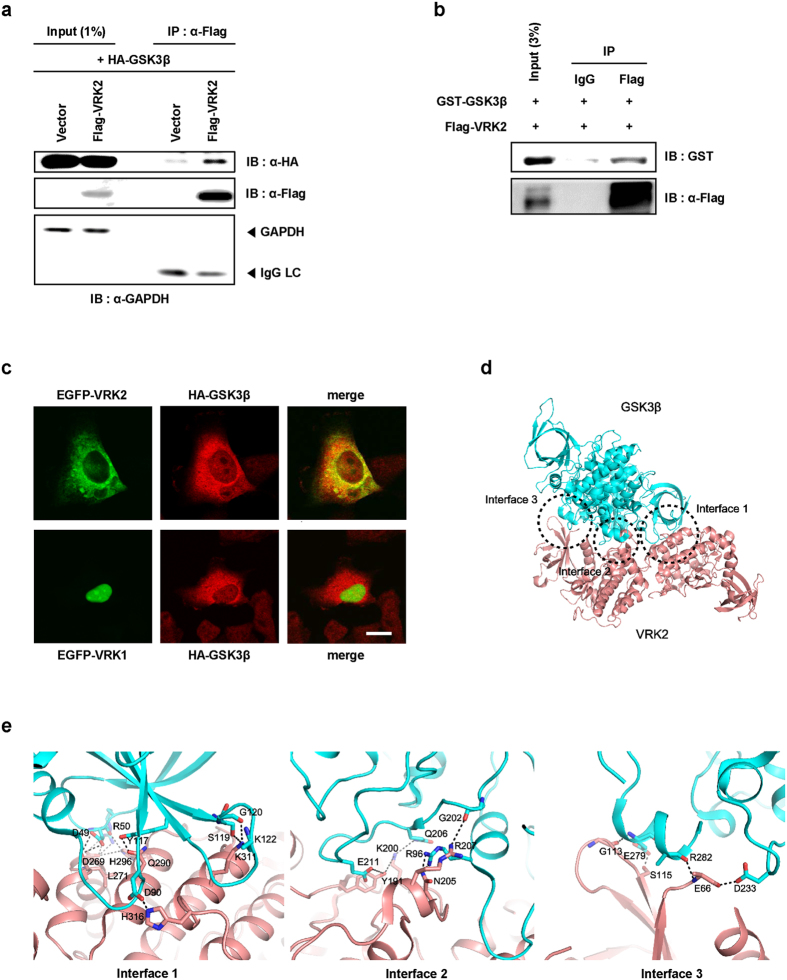
Direct interaction and co-localization of GSK3β with VRK2. **(a)** Whole cell lysates from HEK293T cells were transfected with Flag-Mock or Flag-VRK2 and HA-GSK3β. Twenty-four hours after transfection, whole cell lysates were immunoprecipitated with anti-FLAG antibody and immunoblotted with anti-HA antibody to detect GSK3β. **(b)** Recombinant proteins of Flag-VRK2 and GST-GSK3β fragments in *E. coli*. cultures were immunoprecipitated with Flag antibody or control IgG, and Western blot was performed using anti-GST or anti-Flag antibody. Flag-VRK2 directly bound to GST-GSK3β. **(c)** SH-SY5Y cells were transfected with EGFP-VRK2, or EGFP-VRK1 and HA-GSK3β. The localization of EGFP and HA-GSK3β was observed using fluorescence microscopy. Scale bar, 20 μm. **(d)** The docking model with the highest score for VRK2 (red) interacting with GSK3β (cyan). **(e)** Three binding interfaces between VRK2 and GSK3β. Potential hydrogen bonds are shown as dashed lines. IB, immunoblot; IP, immunoprecipitation.

We previously found that VRK2 indirectly affects the folding of polyQ proteins involved in HD^9^,^14^. To examine whether other proteins are involved in the regulation of VRK2-mediated polyQ protein aggregation, we carried out immunoprecipitation analysis. We found that GSK3β was a VRK2 binding partner. To confirm the interaction between VRK2 and GSK3β, HEK293T cells were transfected with Flag-tagged human VRK2 and HA-tagged GSK3β followed by immunoprecipitation with α-Flag antibody. We found that Flag-VRK2 interacted with HA-GSK3β (Fig. 1a). To verify that GSK3β directly binds to VRK2 *in vitro*, recombinant GSK3β and VRK2 proteins were purified as GST (GST-GSK3β)- and Flag (Flag-VRK2)-fused proteins in *E. coli*, respectively, and immunoprecipitation assays were performed with α-Flag antibody or control IgG. We found that VRK2 directly formed a complex with GSK3β (Fig. 1b). Furthermore, immunocytochemical analysis showed that EGFP-VRK2 colocalized with HA-GSK3β in the same subcellular compartments in SH-SY5Y (Fig. 1c) and HEK293A (Supplementary Fig. S1a) cells.

To study the structural relationship between VRK2 and GSK3β, we performed *in silico* docking analysis. VRK2 and GSK3β structures found in the RCSB Protein Data Bank (PDB) were computationally docked into a 3D model using PatchDock. We found three binding interfaces with high scores in the protein-protein docking model (Fig. 1d). According to the crystal structure of VRK2 (PDB entry: 2V62) and GSK3β (PDB entry: 1I09), residues D269, L271, Q290, H296, K311, and H316 of VRK2 and D49, R50, D90, Y117, S119, G120, and K122 of GSK3β (Interface 1); residues Y191, K200, N205, R207, and D256 of VRK2 and R96, G202, Q206, E211, and E279 of GSK3β (Interface 2); and residues E66, G113, and S115 of VRK2 and D233, E279, and R282 of GSK3β (Interface 3) are crucial for the interaction (Fig. 1e). Furthermore, the electrostatic interaction model of Interface 1 showed that interactions occurred between acidic residues on GSK3β and basic residues on VRK2 (Supplementary Fig. S1b). To understand the influence of surface charges on binding, we conducted point mutations of VRK2. K311, D256 and E66 were selected as key amino acid residues for binding to GSK3β because they have high affinity binding by hydrogen bond pairing. We observed that K311A point mutant (Interface 1), D256A point mutant (Interface 2), E66A point mutant (Interface 3) and K311/D256A/E66A triple mutant had weaker binding affinity for GSK3β compared with wild-type (WT) VRK2 (Supplementary Fig. S1c). Taken together, these results indicate that VRK2 interacts with GSK3β *in vitro*, *in vivo*, and *in silico*.

### GSK3β inhibits VRK kinase activity *in vitro*

**Figure 2:**
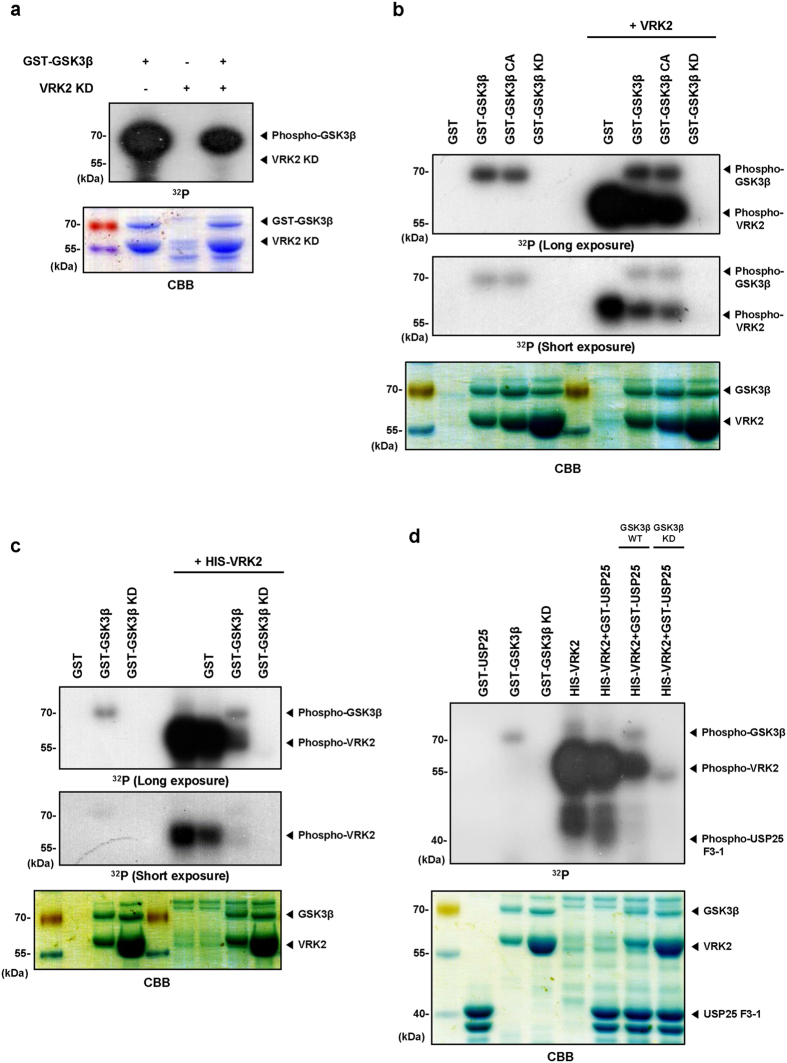
GSK3β inhibits VRK2 in a kinase activity-independent manner. **(a)** Recombinant GSK3β did not phosphorylate VRK2. *In vitro* kinase assay was performed using recombinant GST-GSK3β with inteine-VRK2 KD (kinase-dead mutant with change of lysine61 to alanine in ATP binding sites). Full-length GSK3β was incubated with VRK2 at 30 °C for 30 min, and kinase activity was analyzed by autoradiography and Coomassie blue staining. Arrowheads indicate autophosphorylated GSK3β and trans-phosphorylated VRK2. **(b,c)** Recombinant VRK2 did not phosphorylate GSK3β, and VRK2 kinase activity was inhibited by GSK3β WT or KD (K85A). *In vitro* kinase assay with inteine (or His)-VRK2 and recombinant GST-GSK3β WT, CA (S9A), or KD (K85A) at 30 °C for 30 min. The reaction mixtures were analyzed as in (**a**). The asterisk indicates a nonspecific band. **(d)** VRK2-mediated USP25 fragment phosphorylation was inhibited by GSK3β WT or KD. His-VRK2 and GST-USP25 F3 (fragments 3) derived from transformed *E. coli*. were incubated in the presence or absence of GST-GSK3β WT or KD at 30 °C for 30 min. The reaction mixtures were analyzed as in (**a**). CA, constitutively active; KD, kinase-dead; CBB, Coomassie brilliant blue.

As GSK3β and VRK2 directly interact and are both serine/threonine-protein kinases, we tested whether they phosphorylate each other in an *in vitro* kinase assay. To determine whether VRK2 is a substrate of GSK3β, recombinant His-VRK2 kinase-dead (KD) mutant protein was incubated with recombinant GST-GSK3β protein. We found that GSK3β did not phosphorylate VRK2 (Fig. 2a). Therefore, we repeated *in vitro* kinase assay to determine whether VRK2 phosphorylates GSK3β. We found that VRK2 did not phosphorylate GSK3β or affect its kinase activity (Fig. 2b). However, VRK2 autophosphorylation was decreased in the presence of GSK3β regardless of GSK3β kinase activity (Fig. 2b,c). These results suggest that VRK2 kinase activity is inhibited by GSK3β without protein phosphorylation and that GSK3β could act upstream of VRK2. Our previous study demonstrates that USP25 is phosphorylated and inhibited by VRK2. Among three fragments of UPS25, the fragment containing the regulatory domain between amino acids 655 to 780 from the N-terminal was phosphorylated by VRK2^9^. To clarify whether GSK3β inhibits the effect of VRK2 kinase activity on USP25 phosphorylation, we performed *in vitro* kinase assays with His-VRK2 protein, USP25 fragment, and GST-GSK3β. We found that VRK2-mediated phosphorylation of the USP25 fragment was reduced in the presence of WT or KD GSK3β (Fig. 2d). Furthermore, we confirmed that GSK3β decreased VRK2-mediated histone H3 phosphorylation (Supplementary Fig. S2a). These results suggest that VRK2 kinase activity is inhibited by the presence of GSK3β protein regardless of its kinase activity.

### GSK3β suppresses VRK2-mediated degradation of TRiC

**Figure 3:**
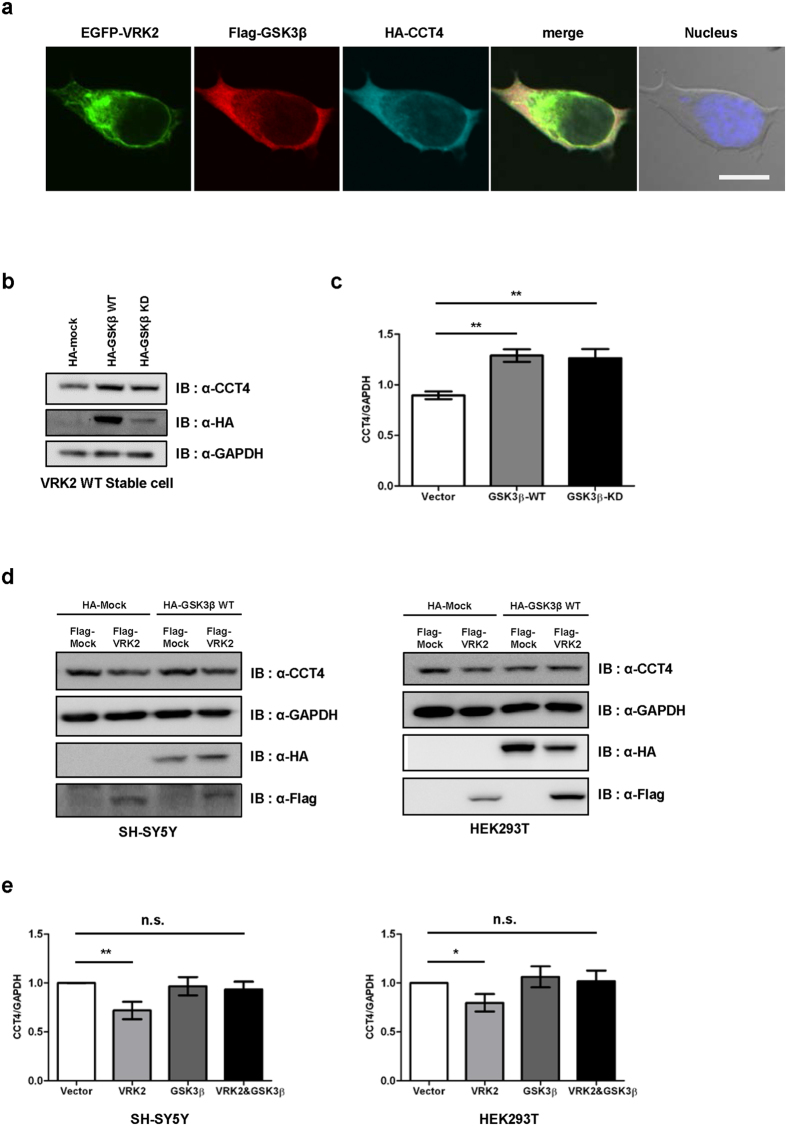
GSK3β inhibits VRK2-mediated degradation of chaperonin TRiC. (**a)** Co-localization of VRK2, GSK3β, and CCT4. EGFP-fused VRK2 (green), Flag-GSK3β, and HA-CCT4 were coexpressed in HEK293A cells and stained with anti-Flag (red) and anti-HA (cyan) antibodies. Hoechst was used for nuclear staining. Scale bar, 10 μm. **(b)** U2OS stable cells expressing VRK2 WT were transfected with HA-GSK3β WT or KD. Western blotting was performed using anti-CCT4 antibody. Overexpression of GSK3β led to increased CCT4 levels. **(c)** Calculated ratio of CCT4/GAPDH levels. One-way ANOVA followed by Tukey’s *post-hoc* tests, **p < 0.01. Data are shown as mean ± SEM (*n* = 4). **(d)** Representative Western blot bands. HA-GSK3β and Flag-VRK2 were coexpressed in SH-SY5Y or HEK293T cells and analyzed by Western blotting. VRK2-mediated degradation of CCT4 levels was decreased by GSK3β WT overexpression. **(e)** Numbers indicate densitometric values determined as CCT4/GAPDH ratios. Values represent mean ± SEM (*n* ≥ 6), *p < 0.05, **p < 0.01, Student’s t-test. n.s., not significant.

The chaperonin TRiC is composed of eight homologous subunits, from CCT1 to CCT8. We previously found that VRK2 facilitates CCT4 polyubiquitination, which downregulates CCT4^14^. Based on the interaction between GSK3β and VRK2 and the observation that GSK3β is an upstream regulator of VRK2, we tested whether GSK3β regulates VRK2-mediated CCT4 downregulation. First, we examined the localization of GSK3β, VRK2, and CCT4. Fluorescence imaging showed that EGFP-VRK2 colocalized with Flag-GSK3β and HA-CCT4 in the same subcellular compartments of HEK293A cells (Fig. 3a). Next, we examined changes in the endogenous level of CCT4 with GSK3β overexpression. For this experiment, we used constitutively VRK2-overexpressing U2OS cells for visualizing prominent polyQ protein aggregates when polyQ-containing protein was overexpressed (Supplementary Fig. S3a). We found that the level of CCT4 protein was significantly increased when WT or KD GSK3β was overexpressed (Fig. 3b,c). Next, we further examined CCT4 protein stability in two different cell lines with overexpression of Flag-VRK2 with or without HA-GSK3β. We found that GSK3β rescued CCT4 protein levels, whereas VRK2 overexpression decreased CCT4 protein levels (Fig. 3d,e). Taken together, these results suggest that GSK3β acts as a positive regulator of CCT4 by inhibiting VRK2.

### GSK3β reduces VRK2-mediated polyQ aggregation

**Figure 4:**
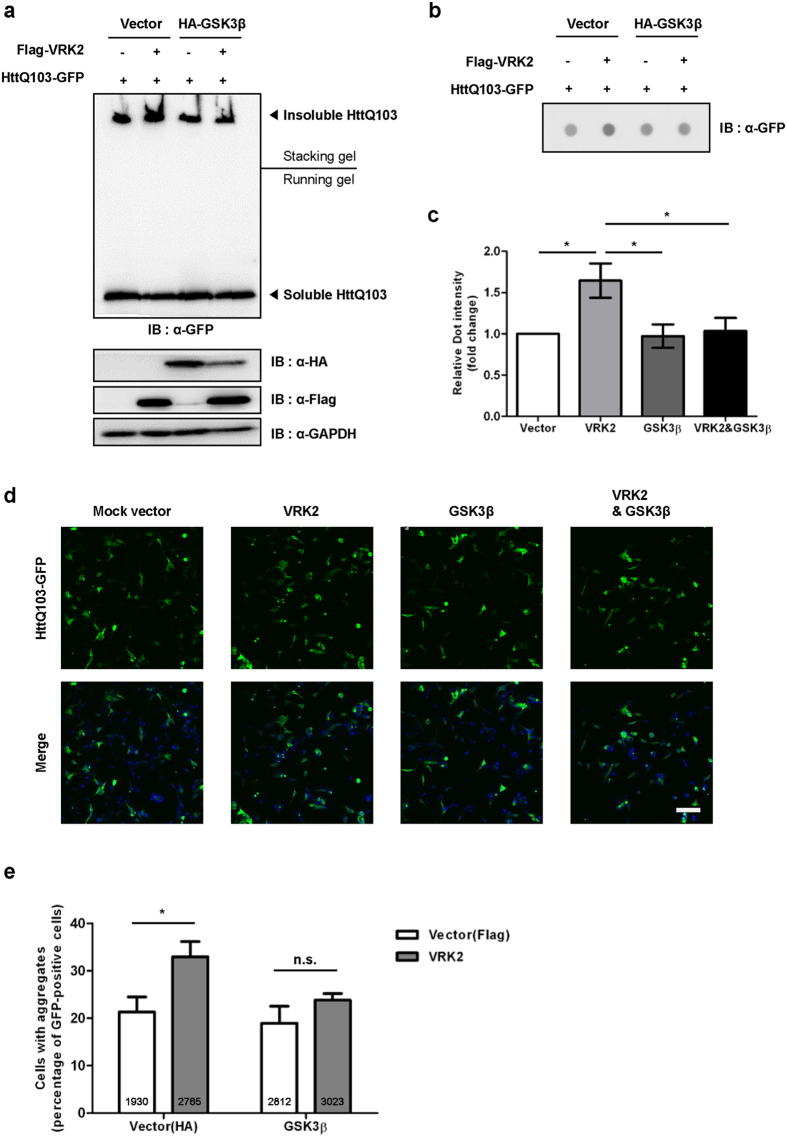
Overexpression of GSK3β suppresses polyQ-expanded Htt aggregation. **(a)** The amount of HttQ103-GFP aggregation was investigated by Western blotting using an anti-GFP antibody. Soluble HttQ103-GFP aggregates are shown in the resolving gel, and SDS-insoluble HttQ103-GFP aggregates are shown in the stacking gel. **(b)** Filter-trap assay for detection of insoluble aggregates was performed after co-transfection of HEK293A cells with HttQ103-GFP, HA-GSK3β, or Flag-VRK2 plasmids. Cell lysates containing equal amounts of protein were filtered on a cellulose acetate membrane, and polyQ-GFP aggregates were detected using an anti-GFP antibody. **(c)** The average value of densitometry in control was set 1. All values are expressed as the mean ± SEM. Statistical comparisons were performed using one-way ANOVA followed by Tukey’s *post-hoc* tests, *p < 0.05, *n *= 6. Values represent mean ± SEM. **(d)** Confocal microscopy was used to assess HttQ103-GFP expression in HEK293A cells transfected with the indicated plasmids. Scale bar, 100 μm. **(e)** The number of cells showing GFP fluorescence with or without aggregates was counted, and the percentage of aggregate-positive cells was calculated. Two-way ANOVA followed by Tukey’s *post-hoc* tests, *p < 0.05, *n* = 4. Data are shown as mean ± SEM. Total counted cell numbers are shown at the bottom. n.s., not significant.

We previously found that VRK2 reduces CCT4 protein levels, resulting in the accumulation of HttQ103 aggregation^14^. To investigate the effect of GSK3β on VRK2-mediated aggregation of polyQ-containing protein, we evaluated polyQ aggregates using a GFP-fused polyQ-expanded Htt fragment. We coexpressed HttQ103-GFP and HA-GSK3β in HEK293A cells with or without overexpression of Flag-VRK2. Interestingly, HttQ103 insoluble aggregates were decreased in HA-GSK3β-overexpressing cells (Fig. 4a). Because GSK3β inhibits degradation of TRiC protein levels by inhibiting VRK2 kinase activity, this suggests that functional TRiC plays a role in inhibiting pathogenic HttQ103 aggregation. In addition to the stabilization of TRiC, the amount of insoluble HttQ103 aggregates was also reduced by overexpression of GSK3β WT or KD (Supplementary Fig. S4a). We further confirmed that GSK3β reduced HttQ103 aggregation by filter-trap assay (Fig. 4b,c, Supplementary Fig. S4b). Consistent with the results of Western blotting and filter-trap assay, HttQ103 aggregates did not increase significantly in GSK3β overexpression with co-overexpression of VRK2 condition while VRK2 alone overexpression increased aggregates (Fig. 4d,e). Collectively, these results suggest that GSK3β-mediated VRK2 inhibition reduces HttQ103 aggregation through the stabilization of chaperonin.

## Discussion

HD is a neurodegenerative disorder caused by an expanded CAG repeat sequence (encoding a poly-glutamine stretch) in the Htt gene^27^, which causes protein aggregation in brain cells and progressive cell death, especially in the striatum and cortex^1^,^2^. Accumulation of mutant Htt protein is toxic and affects important cellular functions such as transcription, axonal transport, synaptic transmission, and mitochondrial function^4^. Mutant Htt interacts with several cellular proteins and sequesters into nuclear aggregates or forms cytoplasmic inclusions that cause progressive neuronal degeneration in HD mouse models. The number of glutamine residues correlates with the severity of HD symptoms, and the pathological hallmark of HD is the formation of polyQ-expanded aggregates^3^,^4^. Therefore, it is important to prevent this process. Therapeutic strategies for HD and other neurodegenerative diseases have focused on elevating molecular chaperones^5^ or activating various cleaning mechanisms, including proteolytic systems^28^ and autophagy^29^. Chaperones promote refolding or degradation of misfolded proteins and prevent protein aggregation. A previous study reports that eukaryotic chaperonin TRiC attenuates the accumulation of Htt-polyQ protein aggregates and their cytotoxicity^6^. Therefore, functional TRiC is involved in decreasing pathogenic HttQ103 aggregate formation.

Recent studies indicate that TRiC directly interacts with and regulates the transcriptional activity of HSF1^30^. Also, GSK3β phosphorylates HSF1 on residue serine 303^31^. Thus, we performed co-immunoprecipitation to test whether GSK3β binds to HSF1 in the presence of VRK2. Binding between GSK3β and HSF1 was not significantly changed by VRK2 overexpression (Supplementary Fig. S5a), suggesting that HSF1 constitutively binds to GSK3β in the presence or absence of VRK2. Next, we examined whether GSK3β-mediated inhibition of VRK2-induced TRiC destabilization affects HSF1 activity by examining transcription levels of the HSF1 target gene hsp70 using real-time quantitative PCR. We found no change in HSF1 transcriptional activity under our experimental conditions (Supplementary Fig. S5b).

We previously found that VRK2 specifically binds to TRiC/CCT subunits, causing chaperonin downregulation through the ubiquitin-proteasome system^14^. This results in a lack of sufficient chaperonin action to mediate proper protein folding in VRK2-overexpressing cells. The level of active VRK2 enzymes may be an important indicator of HD onset and progression. To prove this, alternatively, we investigated VRK2 protein levels in a Huntington’s disease model, R6/2 transgenic mouse, which is a well-studied transgenic animal and most widely used as HD mouse model. The amount of VRK2 protein was relatively increased in the R6/2 transgenic mouse brain lysates compared to wild-type mouse brain lysates at 4 weeks (Supplementary Fig. S6a). In support of this concept, recently, many genome-wide association studies suggest that VRK2 is a risk factor for neurological disorders including schizophrenia^12^,^13^ and epilepsy^11^. VRK2 mRNA levels are upregulated in schizophrenic patient brains compared with normal control brains^12^, and VRK2 gene rs3732136 polymorphism is associated with schizophrenia in the worldwide population^32^. Therefore, genetic variations could affect expression of the VRK2 gene, and malfunctions in VRK2 kinase activity might contribute to susceptibility to neurodegenerative diseases.

In the present study, we demonstrated that GSK3β and VRK2 directly interact and co-localize in cells using *in vitro* and *in vivo* binding assays. Structure-based docking analysis showed the presence of many hydrogen bonds and high-affinity interactions between GSKβ and VRK2. This docking analysis suggests that binding Interfaces 2 and 3 of GSK3β are interacting sites that bind to VRK2 outside of the active site. GSK3β is positioned near the catalytic domain of VRK2, and the binding of GSK3β to VRK2 altered protein flexibility in regions involved in substrate binding and turnover, suggesting their importance to kinase activity. Thus, GSK3β may inhibit VRK2 catalytic activity by disrupting its flexibility. The inhibition of VRK2 catalytic activity by GSK3β may also inhibit VRK2-induced degradation of TRiC, which could suppress polyQ-expanded Htt aggregation. However, a previous study demonstrates that GSK3β inhibitor protects polyglutamine protein-induced cell death but had inconsistent effects on the number of Htt aggregates in cells^33^. GSK3β inhibitor also has variable effects in a transgenic mouse model of HD^34^ and has beneficial effects when combined with other drugs^35^,^36^. However, the main reason why GSK3β inhibitors do not have reliable effects may be due to markedly reduced levels and activity of GSK3β in the striatum and other brain regions in R6/1 HD mouse models and the frontal cortex of postmortem HD patients^25^. Our findings support previous observations of decreased levels and reduced activity of GSK3β in HD mouse models and postmortem human brains. Therefore, the level of GSK3β in the brain may be critical for controlling HD progression.

**Figure 5:**
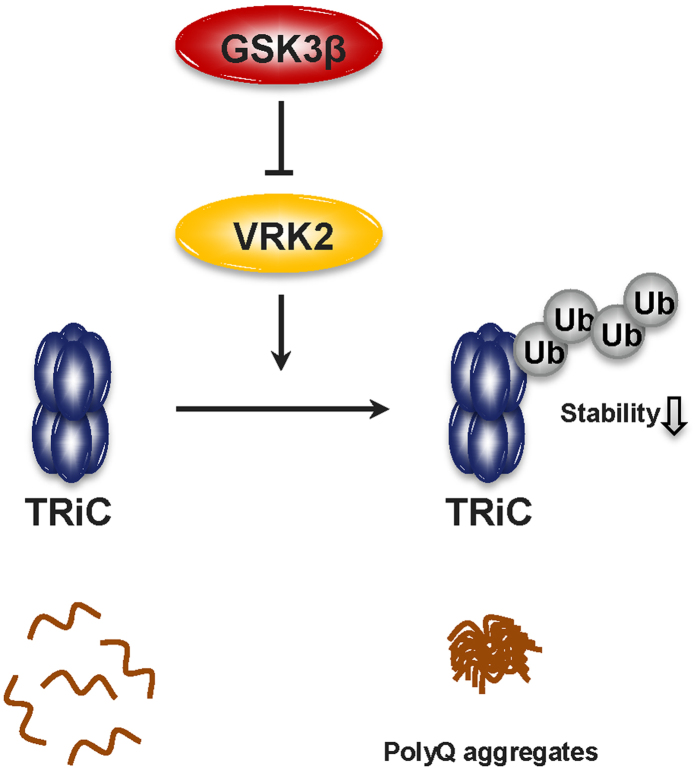
Diagram of GSK3β-VRK2 signaling pathway related to regulation of TRiC/CCT. The TRiC/CCT complex reduces the formation of polyQ protein aggregates. VRK2 affects TRiC protein degradation by regulating the stability of CCT4 subunits. We found that GSK3β interacted with VRK2 and inhibited VRK2 catalytic activity, which reduced HttQ103 aggregation through increased CCT4 stability.

In conclusion, we propose that GSK3β has positive effects on HD by regulating VRK2 and the stability of chaperonin TRiC. GSK3β appears to inhibit VRK2 kinase activity via protein-protein interaction in a kinase activity-independent manner, thereby reducing ubiquitination-proteosomal degradation of TRiC and suppressing polyQ protein aggregation (Fig. 5). Our results could aid in the development of efficient ways for preventing or treating HD by regulating GSK3β levels.

## Materials and Methods

### Plasmids

All plasmids used in this study were described in earlier studies^9^,^14^. VRK2 kinase-dead mutant was generated by site-directed mutagenesis (Lys61 to Ala). Flag-hVRK2-mutants (K311A (Interface 1), D256A (Interface 2), and E66A (Interface 3)) were amplified from Flag-hVRK2-WT plasmid using a forward primer (5′-AAGCGGCCGCAATGCCACCAAAAAGAAATG-3′) and reverse primer (5′-AATCTAGATCAGAGAAAAAATAAAGCAAGAA-3′). The first round of PCR, using forward and reverse primer (5′-GGTTCAAAATTGCCTTGAGGGC-3′ for Interface 1, 5′-AGCCACAGGTGCCTTCAGGTT-3′ for Interface 2, and 5′-CGGGCCATTTGCTTGATATTC-3′ for Interface 3) and another forward (5′-GCCCTCAAGGCAATTTTGAAC-3′ for Interface 1, 5′-AACCTGAAGGCACCTGTGGCT-3′ for Interface 2, and 5′-GAATATCAAGCAAATGGCCCG-3′ for Interface 3) and reverse primer, created two fragments. The two PCR products were mixed together for a second round of PCR. The full-length coding sequence of hVRK2 was digested with NotI/XbaI and cloned into pFlag-CMV2 (Sigma, St. Louis, MO) control vector. The GSK3β constructs were gift from Sang Ki Park (POSTECH, Pohang, South Korea). The HA-tagged cDNA of human GSK3β wild-type and two mutants, catalytically inactive GSK3β (GSK3β K85A) and constitutively active GSK3β (GSK3β S9A), were cloned into pGEX-4T-3 (Amersham, Piscataway, NJ).

### Antibodies

Antibodies used in this study included: anti-glyceraldehyde-3-phosphate dehydrogenase (GAPDH) and anti-GST (Santa Cruz Biotechnology, Santa Cruz, CA, USA), anti-HA (Bethyl, Montgomery, TX, USA), anti-CCT4 (Abcam, Cambridge, United Kingdom), and anti-FLAG (Sigma, St. Louis, MO). All fluorescence-conjugated secondary antibodies were from Invitrogen.

### Cell culture and transfection

HEK293A, HEK293T, U2OS, and SH-SY5Y cells were propagated in Dulbecco’s Modified Eagle’s Medium or Minimum Essential Media supplemented with 10% fetal bovine serum and 100 U/ml each of penicillin G and streptomycin in 5% CO_2_ at 37 °C. For transient transfection with plasmids, lipofectamine 2000 (Invitrogen, Carlsbad, CA, USA) or Microporator (Invitrogen) was used according to the manufacturer’s instructions.

### Biochemical methods

Cell extract and protein preparation, immunoprecipitation, and Western blotting were performed as previously described^9^,^14^. Protein bands were visualized using a SUPEX ECL solution kit (Neuronex, Daegu, South Korea) and a LAS-4000 chemiluminescence detection system (Fujifilm, Tokyo, Japan).

### *In vitro* kinase assay

*In vitro* kinase assay with recombinant proteins was performed as previously described^9^.

### Filter-trap assay

Cells were lysed in RIPA buffer, and sonicated lysates were diluted in 1% SDS-PBS and boiled at 95 °C for 5 min. The membrane was pre-equilibrated with 1% SDS in PBS. Proteins were loaded onto a 0.22-μm nitrocellulose membrane. A dot blotter apparatus (Bio-Rad, Hercules, CA) was used for the application of samples, and anti-GFP antibody (Santa Cruz, CA) was used for detection.

### Fluorescence microscopy

Transfected cells were maintained for 24–48 h, fixed with 4% paraformaldehyde for 20 min, and immunostained with appropriate primary and fluorescence-conjugated secondary antibodies. Coverslips were mounted onto slides using Fluoromount (Sigma). All images were obtained using a laser scanning confocal microscope (model FV1000; OLYMPUS), and FV10-ASW2.0 fluoviewer software was used for image analysis.

### Structural modeling and analysis

PatchDock was used to study the structural relationship between GSK3β and VRK2^37^. According to the crystal structure of GSK3β (PDB entry: 1I09)^38^ and VRK2 (PDB entry: 2V62)^39^, the best structure for docking was selected and further analyzed using PyMOL.

### Establishment of stable cell lines

Stable cell lines were generated by G418 selection. Briefly, pNTAP-mock or pNTAP-hVRK2 vector was transfected into the normal U2OS cell line using electroporation and incubated for 24 h. Cells were selected by 800 μg/ml G418 with media changes every 2 days until single colonies formed. After single colony isolation, stable cells were maintained with 200 μg/ml G418-containing media.

### Quantitative real-time reverse transcription PCR

Total RNA was isolated using TRI reagent (Molecular Research Center, Cincinnati, OH) and reverse transcribed using ImProm-II (Promega) according to the manufacturer’s instructions. For detection and quantification, the StepOnePlus real-time PCR system (Applied Biosystems) was used.

### Ethics statement

Approval of the study protocol was obtained from the Pohang University of Science and Technology Institutional Animal Care and Use Committee (POSTECH IACUC) (Approval ID: 2013-03-004). All animal experiments were carried out according to the provisions of the Animal Welfare Act, PHS Animal Welfare Policy, and the principles of the NIH Guide for the Care and Use of Laboratory Animals. All mice were maintained under conventional conditions at the POSTECH animal facility under institutional guidelines.

### Statistical analysis

Quantitative data are shown as mean ± standard error of the mean (SEM). Statistical analyses were performed using Student’s t-test and one-way analysis of variance (ANOVA), two-way ANOVA and *post-hoc* Tukey’s multiple comparison tests using GraphPad software.

## Additional Information

**How to cite this article**: Lee, E. *et al*. Glycogen synthase kinase 3β suppresses polyglutamine aggregation by inhibiting Vaccinia-related kinase 2 activity. *Sci. Rep*. **6**, 29097; doi: 10.1038/srep29097 (2016).

## Supplementary Material

Supplementary Information
