# Supplementary material for: Glycogen synthase kinase 3β suppresses polyglutamine aggregation by inhibiting Vaccinia-related kinase 2 activity
Source: Sci Rep. 2016 Jul 5;6:29097. doi: 10.1038/srep29097 (PMC4932512; doi:10.1038/srep29097)
Supplement: Supplementary Information [file srep29097-s1.pdf]

Supplementary information for

Glycogen synthase kinase 3β suppresses polyglutamine aggregation by inhibiting Vaccinia-related kinase 2 activity

Eunju Lee[1†], Hye Guk Ryu[2†], Sangjune Kim[2, 3, 4], Dohyun Lee[2], Young-Hun Jeong[2] and Kyong-Tai Kim[1, 2,*]

[1]Division of Integrative Biosciences and Biotechnology, [2]Department of Life Sciences, Pohang University of Science and Technology, Pohang 790-784, Republic of Korea; [3]Neuroregeneration and Stem Cell Programs, Institute for Cell Engineering, [4]Department of Neurology, The Johns Hopkins University School of Medicine, Baltimore, Maryland 21205, United States of America

† The authors contributed equally.

***Corresponding author information:**

Kyong-Tai Kim, Ph.D., Professor

Division of Integrative Biosciences and Biotechnology, POSTECH

Hyoja Dong, Pohang, Gyeongbuk, Korea 790-784

Tel: 82-54-279-2297

E-mail: ktk@postech.ac.kr

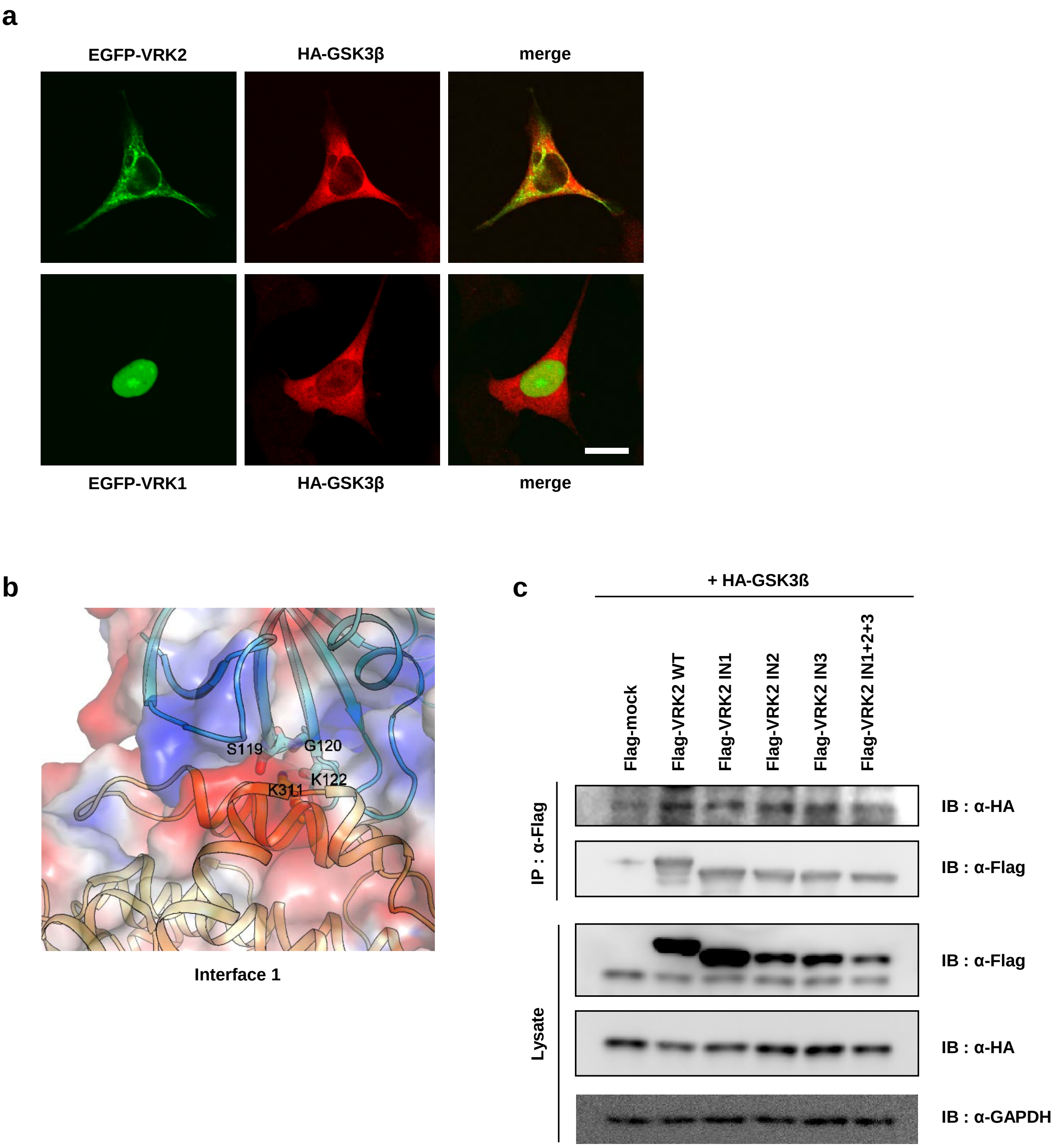

Supplementary Figure S1. VRK2 specifically interacts with GSK3β. (a) HEK293A cells were transfected with EGFP-VRK2, or EGFP-VRK1 and HA-GSK3β. Nuclei were stained with Hoechst, and the localization of VRK2, or VRK1 and GSK3β was observed with fluorescence microscopy. Scale bar, 20 μm. **(b)** Electrostatic interactions model of Interface 1 between GSK3β (cyan) and VRK2 (yellow) using PatchDock. The negatively charged residues of GSK3β interacted with the oppositely charged residues of VRK2. VRK2 K311 forms hydrogen bonds with S119, G120 and K122 in GSK3β. **(c)** Immunoprecipitation analysis with anti-Flag antibodies in HEK293A cells expressing either wild-type Flag-VRK2 or Flag-VRK2-mutants (K311A (IN1), D256A (IN2), E66A (IN3) or K311/D256A/E66A triple mutant (IN1+2+3)) with HA-GSK3β. IN, Interface.

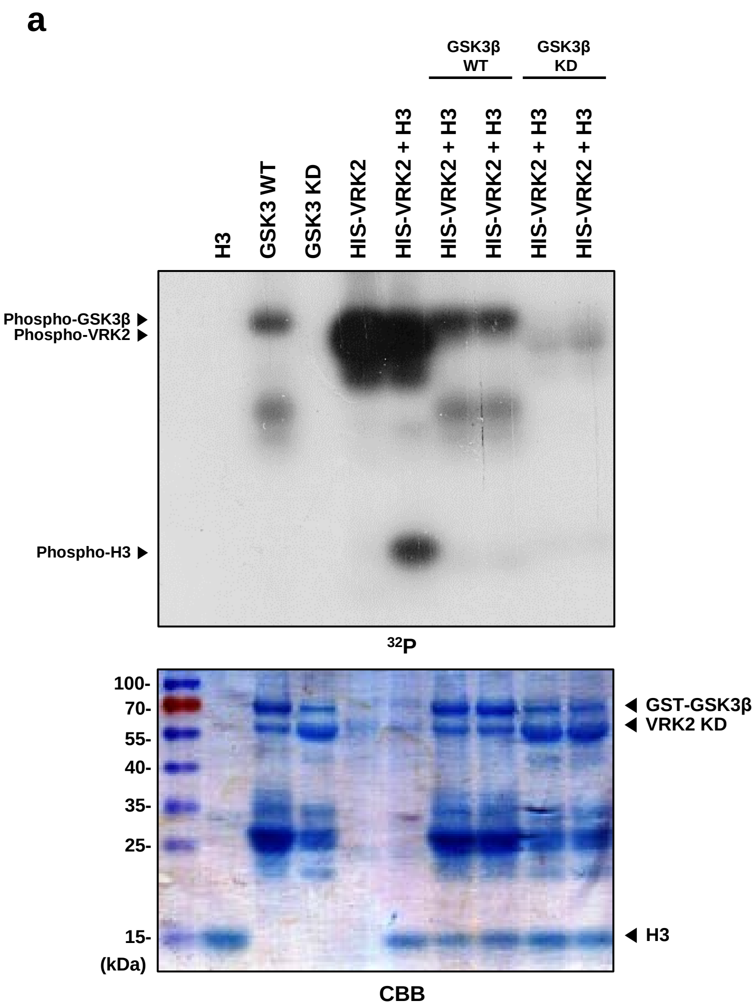

Supplementary Figure S2. GSK3β inhibits VRK2 kinase activity. (a) VRK2-mediated Histone H3 phosphorylation is inhibited by GSK3β WT or KD *in vitro* (top panel). The Coomassie brilliant blue (CBB)-stained membrane is shown as a loading control (bottom panel). WT, wild type (kinase active); KD, kinase dead; CBB, coomassie brilliant blue.

a

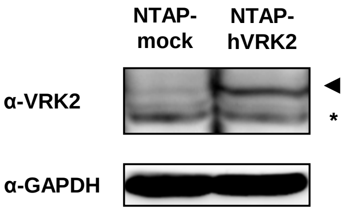

Supplementary Figure S3. Generation of stable VRK2-overexpressing cells. (a) Stable VRK2-overexpressing U2OS cell lysates were subjected to immunoblotting with the indicated antibodies. The arrowhead indicates NTAP-hVRK2, and the asterisk indicates endogenous VRK2.

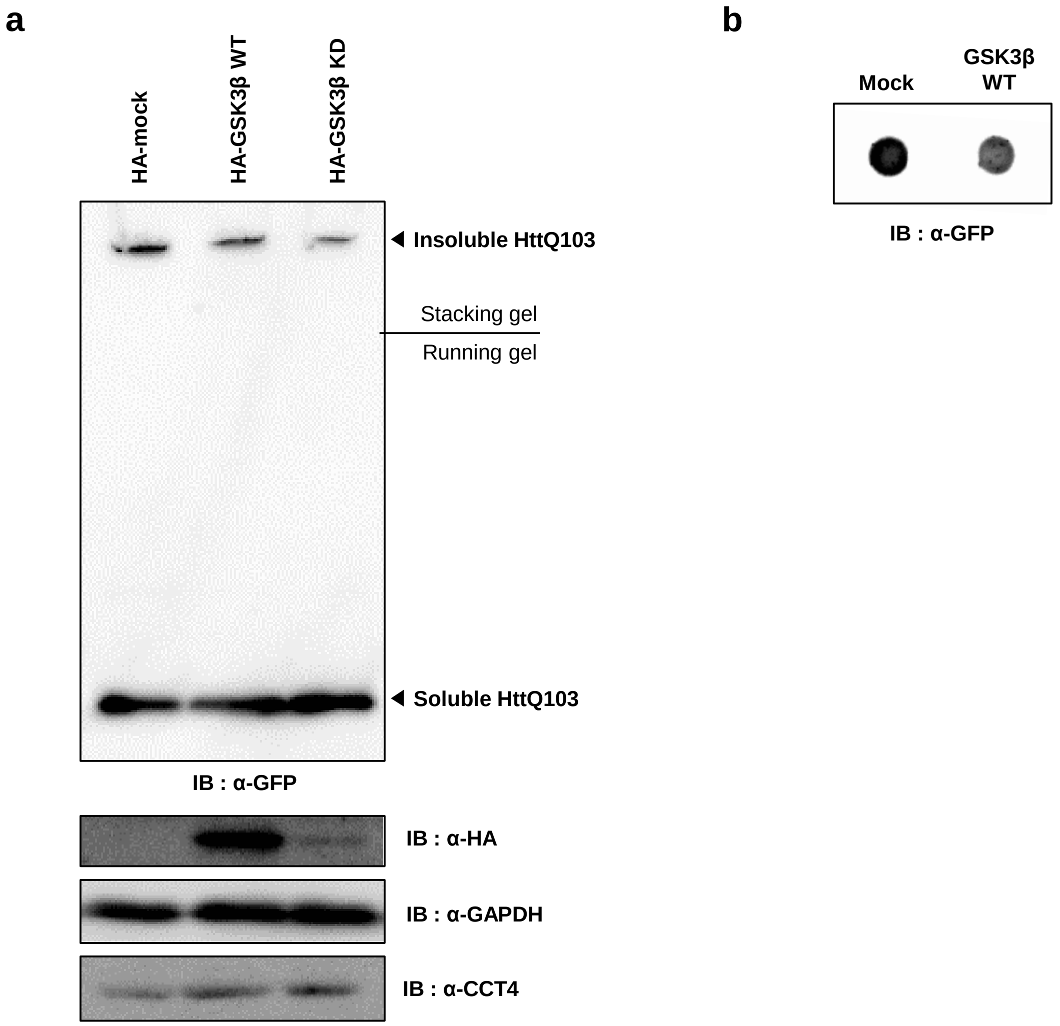

Supplementary Figure S4. GSK3β enhances the chaperone activity of TRiC and reduces polyQ-expanded Htt aggregation. (a) HttQ103-GFP with HA-GSK3β WT or HA-GSK3β KD was coexpressed in U2OS stable cell lines expressing VRK2 WT and analyzed by Western blotting using an anti-GFP antibody to detect polyQ aggregates. **(b)** Analysis of HttQ103-GFP aggregates using filter-trap assay. VRK2-overexpressing U2OS cells were cotransfected with a construct of HttQ103-GFP and HA-GSK3β WT. Cell lysates containing equal total amounts of protein were filtered on a cellulose acetate membrane, and polyQ-GFP aggregates were detected using an anti-GFP antibody.

a

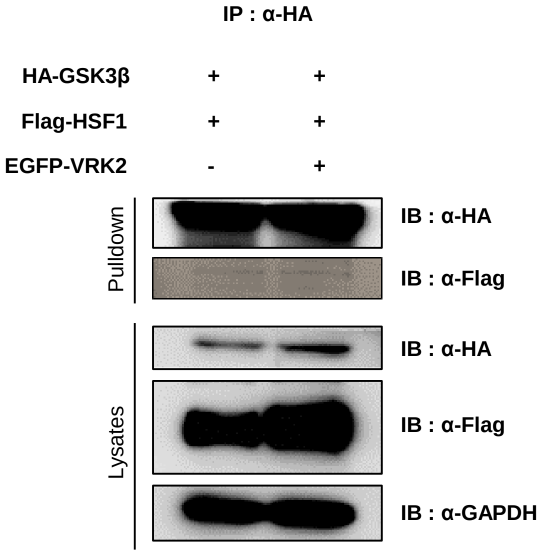

b

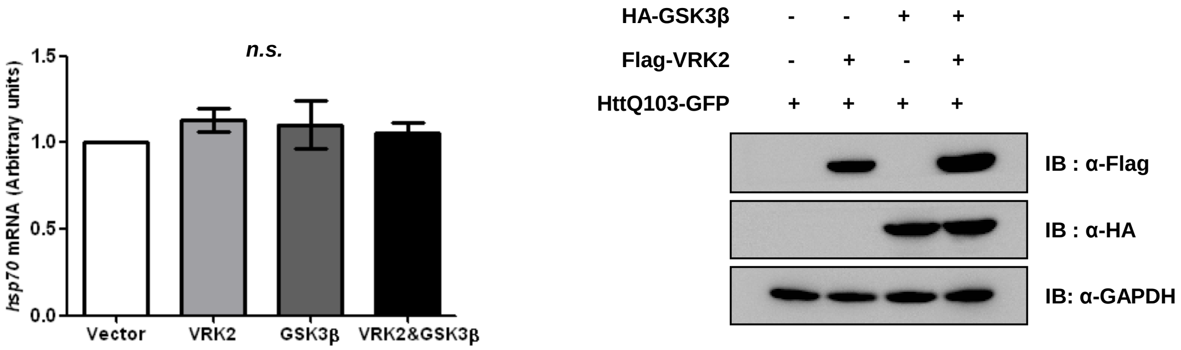

Supplementary Figure S5. VRK2 did not affect binding of GSK3β to HSF1 or *hsp70* mRNA levels. (a) HEK293T cells were transfected with HA-GSK3β and Flag-VRK2 with or without EGFP-VRK2. After 24 h of expression, HEK293T cells were harvested, and cell lysates were subjected to immunoprecipitation with HA antibody and sequential immunoblotting with the indicated antibodies. **(b)** *hsp70* mRNA levels were examined by quantitative real-time RT-PCR and normalized to *gapdh* mRNA levels. n.s., not significant.

a

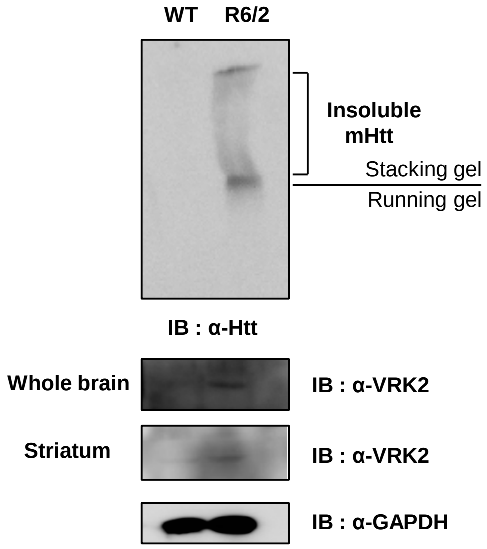

Supplementary Figure S6. VRK2 protein levels were increased in transgenic mouse model for Huntington's disease (R6/2). (a) Whole brain and striatum homogenates of R6/2 transgenic mice were analyzed. The aggregates of mutant Htt were detected with antibodies against huntingtin in the stacking gel.
